# Genome-Wide Identification of KANADI1 Target Genes 

**DOI:** 10.1371/journal.pone.0077341

**Published:** 2013-10-14

**Authors:** Paz Merelo, Yakun Xie, Lucas Brand, Felix Ott, Detlef Weigel, John L. Bowman, Marcus G. Heisler, Stephan Wenkel

**Affiliations:** 1 European Molecular Biology Laboratory (EMBL), Heidelberg, Germany; 2 School of Biologlical Sciences, Sydney University, Sydney, Australia; 3 Center for Plant Molecular Biology, University of Tübingen, Tübingen, Germany; 4 School of Biological Sciences, Monash University, Melbourne, Australia; 5 Max-Planck-Institute for Developmental Biology, Tübingen, Germany; Instituto de Biología Molecular y Celular de Plantas, Spain

## Abstract

Plant organ development and polarity establishment is mediated by the action of several transcription factors. Among these, the KANADI (KAN) subclade of the GARP protein family plays important roles in polarity-associated processes during embryo, shoot and root patterning. In this study, we have identified a set of potential direct target genes of KAN1 through a combination of chromatin immunoprecipitation/DNA sequencing (ChIP-Seq) and genome-wide transcriptional profiling using tiling arrays. Target genes are over-represented for genes involved in the regulation of organ development as well as in the response to auxin. KAN1 affects directly the expression of several genes previously shown to be important in the establishment of polarity during lateral organ and vascular tissue development. We also show that KAN1 controls through its target genes auxin effects on organ development at different levels: transport and its regulation, and signaling. In addition, KAN1 regulates genes involved in the response to abscisic acid, jasmonic acid, brassinosteroids, ethylene, cytokinins and gibberellins. The role of KAN1 in organ polarity is antagonized by HD-ZIPIII transcription factors, including REVOLUTA (REV). A comparison of their target genes reveals that the REV/KAN1 module acts in organ patterning through opposite regulation of shared targets. Evidence of mutual repression between closely related family members is also shown.

## Introduction

Plants achieve their final shoot architecture through the proper positioning of lateral organs such as leaves and flowers. In part this is mediated by the polar transport of the plant hormone auxin to specific locations, which then triggers organ initiation at these sites. The subsequent differentiation of organ progenitor cells into more specialized cell types results in highly organized tissues made up of many distinct cell types. The KAN subclade of the GARP family of transcription factors, as well as the set of class III homeodomain leucine zipper (HD-ZIPIII) transcription factors, play important roles in polarity-associated patterning processes. These transcription factors are key determinants in embryo, shoot and root patterning and during vegetative growth regulate several organ polarity processes [1-15]. In particular, during leaf development these two gene families have been shown to act antagonistically to maintain a stable abaxial/adaxial boundary (the boundary between the lower and upper side of the leaf) that is necessary for proper leaf blade growth. Here, the four members of the KAN group (*KAN1-4*) are required for abaxial cell fate, whereas the HD-ZIPIII genes, including *PHABULOSA* (*PHB*), *PHAVOLUTA* (*PHV*) and *REV*, promote adaxial cell identity in organ primordia [1,3,4,7,12-14]. 

Genetic studies have identified additional regulatory factors specifying the abaxial/adaxial sides of the leaf. The *ASYMMETRIC LEAVES2* (*AS2*) gene, a LOB domain–containing plant-specific protein, and the *ASYMMETRIC LEAVES1* (*AS1*) gene, a MYB domain transcription factor, are involved in the development of a symmetrical expanded lamina, and act to promote adaxial (upper leaf) fate in this regulatory network [16-18]. On the opposite side, members of the YABBY (YAB) gene family, such as *FILAMENTOUS FLOWER* (*FIL*), *YAB3*, *YAB5* and *YAB2*, and two *AUXIN RESPONSE FACTOR* genes (ETTIN (ETT)/*ARF3* and *ARF4*), specify abaxial (lower leaf) cell fate [19-23]. In addition to this set of transcription factors, small RNAs have also been found to play crucial roles in the establishment of organ polarity. *HD-ZIPIII* factors are targeted by *microRNAs 165/166*, which therefore act as abaxial determinants [24-27]. The *ARF3* and *ARF4* genes are controlled by the ta-siRNAs *ta-siR2141* and *ta-siR2142* (also referred as *ta-siR-ARFs*), thus implicating *tasiR-ARFs* as important adaxial regulators [27,28]. 

Genetic analysis indicates that some of these genes act antagonistically: loss-of-function mutations in genes promoting adaxial development typically produce an abaxialized phenotype that is accompanied by the expanded expression of abaxial genes, whereas loss-of-function mutations in abaxial genes produce an adaxialized phenotype that correlates with the expanded expression of adaxial genes. Transgenes or mutations that cause ectopic expression of these genes, usually lead to phenotypes opposite to that of the loss-of-function mutations. The antagonistic relationship between the adaxial and abaxial transcription factors could be mediated by direct cross regulation of each other's expression, or alternatively but not mutually exclusively, via opposite effects on common downstream targets of biochemical processes, both of which have been postulated [7,10,29-31]. One strategy to understand how transcription factors mediate their developmental functions is to identify the genes they directly regulate. In this study, we focus mainly on identifying KAN1 targets and, in addition, define potential shared targets between the abaxial factor KAN1 and the adaxial-fate promoting factor REV. 

Up to now, only a small number of REV and KAN1 target genes have been reported. For instance, the *LITTLE ZIPPER* (*ZPR*) genes have been proposed as direct REV targets since they are transcriptionally up-regulated by REV and other HD-ZIPIII transcription factors. Furthermore, ZPR proteins interact with and repress HD-ZIPIII activity, forming a negative feedback loop [32,33]. Recently, we demonstrated that REV acts upstream of several class II HD-ZIP transcription factors (*HAT2*, *HAT3*, *ATHB2/HAT4* and *ATHB4*) involved in shade signaling and leaf development [29,34], and the auxin biosynthetic enzymes TAA1 and YUCCA5 (YUC5). Expression of *HAT2*, *TAA1* and *YUC5* is reduced significantly by dexamethasone (DEX) in inducible KAN1 overexpression lines (*35S::FLAG-GR-KAN1*), indicating that at least one way to establish the leaf adaxial-abaxial pattern by the REV/KAN module is through the opposite regulation of shared target genes [29]. In addition, KAN activity has been proposed to negatively regulate PIN expression, and hence auxin movement, based on the ectopic expression of PIN1 in *kan* loss-of-function alleles, and the rapid down-regulation of *PIN1* expression in response to induction of ectopic KAN1 activity [7,10]. It is not known whether KAN regulation is direct or indirect, but also suggests opposing actions of KAN and HD-ZIPIII on regulation of auxin biology. The adaxial factor *AS2* is the best characterized target gene of KAN1, which represses the transcription of *AS2* in abaxial tissue [17,18,31]. Mutation of a single nucleotide in a KAN1 binding site in the *AS2* promoter causes ectopic *AS2* expression in the abaxial domain, resulting in an adaxial phenotype. Furthermore, it has been shown that the abaxial expression of *KAN1* is mediated directly by AS2 [31]. Based on these results, it has been proposed that KAN1 acts as a transcriptional repressor, and that mutual repression between KAN1 and AS2 contributes to the proper establishment of abaxial/adaxial polarity in plants.

Here, we provide a set of potential target genes of the KAN1 transcription factor identified through a combination of chromatin immunoprecipitation/deep sequencing (ChIP-Seq) and genome-wide transcriptional profiling using tiling arrays. Our dataset shows a strong over-representation of genes involved in the regulation of organ development as well as in the response to hormonal stimuli. In addition, the *cis*-element ‘VGAATAW’ has been identified to be enriched in the ChIP-seq dataset providing the first information about the KAN1-binding site. This *cis*-element is also present in the promoter of the KAN1 target gene *AS2* and it has been shown to be recognized by KAN1 [31], validating our ChIP-seq analysis. Finally, the identification of genes potentially dually regulated by the REV/KAN1 module enables future elucidation of different genetic networks underlying the action of these antagonistic factors. 

## Materials and Methods

### Plant material and treatments

For efficient chromatin immunoprecipitation, transgenic *35S::FLAG-GR-KAN1* plants were used [29]. The glucocorticoid receptor (GR) was cloned in frame with the FLAG epitope in the pJAN33 vector using the KpnI restriction site [35]. Therefore, these transgenic plants can be treated with dexamethasone (DEX), inducing the transition of the chimeric FLAG-GR-KAN1 protein from the cytoplasm to the nucleus, where it can bind to DNA to regulate its downstream targets. In order to achieve equal distribution and uptake of DEX, *35S:FLAG-GR-KAN1* plants were grown in liquid culture for 10 days and induced with 25µM DEX for 45 minutes prior to chromatin-immunoprecipitation. As a control, we used wild type Columbia (Col-0) plants.

### ChIP-sequencing and ChIP analysis

Chromatin extraction and immunoprecipitation (ChIP) were carried out as described by Brandt et al. (2012) [29]. In total, we constructed one control library (Col-0) and two ChIP-Seq libraries for *35S:FLAG-GR-KAN1* using the Illumina® TruSeq® ChIP Sample Preparation Kit, according to the manufacturer’s protocol. For library preparation indexing adapters were ligated to the ends of the DNA fragments (AR003 for Col-0 library and AR011 and AR027 for *35S:FLAG-GR-KAN1* libraries). Indexed libraries were subsequently subjected to deep sequencing using the Illumina HiSeq instrument. The Illumina sequencing and data analysis were performed as described by Yant and colleagues (2010) [36], with the exception that the number of duplicate sequence reads was heuristically reduced prior to further analysis. This ChIP-Seq experiment resulted in the identification of 17402 positions in the *Arabidopsis* genome being enriched in *35S::FLAG-GR-KAN1* plants compared with Col-0 plants. ChIP-Seq raw data obtained in this study are available at the Gene Expression Omnibus database under series accession number GSE48081.

### Tiling arrays

To examine genome-wide effects of high levels of KAN1 activity, we used ubiqutious expression of a steroid-dependent KAN1 variant, *35S:KAN1-GR* [25]. KAN1 protein activity was induced by growing plants on 0.5x MS plates and submerging seedlings in 10 µM dexamethasone 21-acetate solution for 5 minutes. RNA was collected at three time points: 0 minutes (pre-induction) and 80, and 160 minutes post-induction. A total of 20-30 µg total RNA per sample using the RNeasy® Plant mini Kit (Qiagen, Valencia, CA, USA) was converted into a labeled probe for hybridization to *Arabidopsis* Tiling 1.0 Arrays (Affymetrix) at the Australian Genome Research Facility (The Walter and Eliza Hall Institute of Medical Research, Melbourne, Australia). The results were then calibrated and pooled per time point (2-3 biological replicates per time point) according to the tiling 1.0 array manual, and the resulting .chp files where loaded versus control into the Integrated Genome Browser (version 6.7) software for analyses [37]. The transcriptional changes from baseline were graphically assessed using selected threshold values and candidates with consistent up/down regulation along the full ORF/length of the predicted expressed sequence were identified. 

### Semi-quantitative PCRs (sqPCR) and quantitative real-time PCRs (qPCRs)

To test the *35S:KAN1-GR* line used for the tiling array experiment, *AS2* and cyclophilin were assessed as positive and negative controls, respectively, by sqPCR.

RNA was extracted from 15 day old seedlings grown on MS medium and after 80 minutes of dexamethasone treatment using the RNeasy® Plant mini Kit (Qiagen, Valencia, CA, USA). 1 µg of purified RNA was treated with DNAse RQ1 (Promega, Madison, WI, USA) and reverse transcribed using PrimeScript^TM^ Reverse Transcriptase (TaKaRa Biotech) for sqPCR. The sqPCR was performed with three biological replicates and visualized on 1.5% agarose gels using electrophoresis [10].

To analyze the gene expression of *ATHB8*, RNA was isolated from 10 day old Col-0 and transgenic *35S:FLAG-GR-KAN1* seedlings after 4 hours of DEX induction. Glyceraldehyde 3-phosphate dehydrogenase (GADPH) was used as a reference gene to evaluate the amounts of mRNA (Figure S2). Real-time PCR experiments were performed as described by Brandt et al. (2012) [29]. 

## Results

### Identification of direct KAN1 target genes using ChIP-Seq

**Figure 1:**
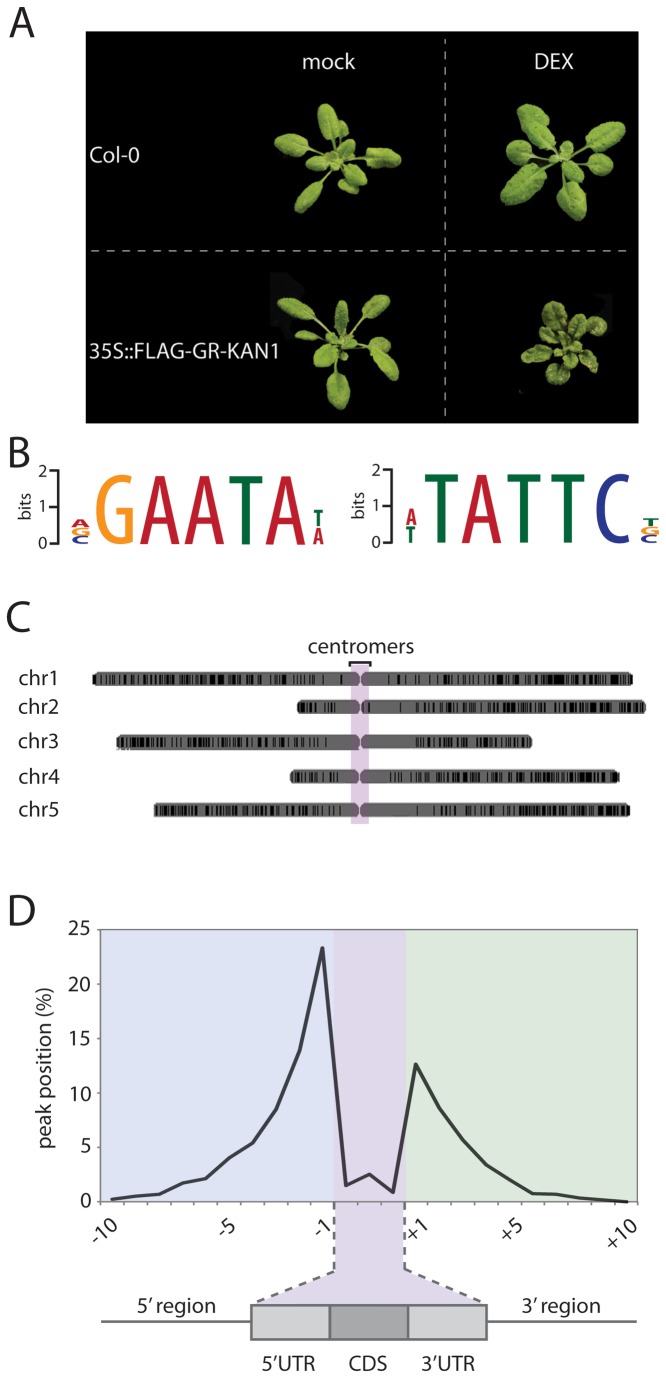
Identification of KAN1 target genes. **A**) Constructing an inducible KAN1 expression system. **B**) Sequence logos for the *cis*-element, forward and reverse orientation, enriched in the ChIP-Seq dataset **C**) Distribution of KAN1 binding sites across the five *Arabidopsis* chromosomes. **D**) Location of peaks identified by ChIP-Seq. About 25% of all peaks are located in the first 1000bp upstream of the transcriptional start site.

To better understand processes downstream of KAN1 action, we constructed transgenic plants over-expressing KAN1 fused to the rat glucocorticoid receptor carrying an additional FLAG-epitope (*35S:FLAG-GR-KAN1*). After growing these plants on soil until the first true leaves were visible, the plants were sprayed once a day for one week with 25µM DEX solution. This treatment resulted in the development of partially abaxialized leaves with drastically reduced petioles (Figure 1A), whereas untreated control plants showed no mutant phenotype. In order to achieve equal distribution and uptake of DEX, *35S:FLAG-GR-KAN1* plants were grown in liquid culture for 10 days and induced with 25µM DEX for 45 minutes prior to chromatin-immunoprecipitation. As a control, we isolated chromatin from Col-0 wild-type plants. One Illumina control library and two ChIP-Seq libraries for *35S:FLAG-GR-KAN1* were sequenced. After filtering for read quality, sequencing reads were mapped to the *Arabidopsis* genome (TAIR10), resulting in the identification of 17402 peaks that were enriched in two independent ChIP-Seq experiments over the control sample. We subsequently limited our analysis to peaks showing at least three-fold enrichment. This dataset contains 4183 KAN1 bound regions. From a MEME-ChIP analysis (http://www.meme.sdsc.org) a VGAATAW motif was identified in 1802 of the 4183 regions (Figure 1B), corresponding to 3151 genes potentially regulated by KAN1 (see Dataset S1). These loci were equally distributed over the five *Arabidopsis* chromosomes, with a lack of enriched peaks in the centromeric regions (Figure 1C). A further analysis of the distribution of the peaks relative to the gene models revealed that the majority of binding sites were located within 1.0 kb upstream of the transcriptional start site (about 24%) or 1.0 kb downstream of the coding region (about 11%). Peaks were underrepresented in gene coding regions (Figure 1D).

**Figure 2:**
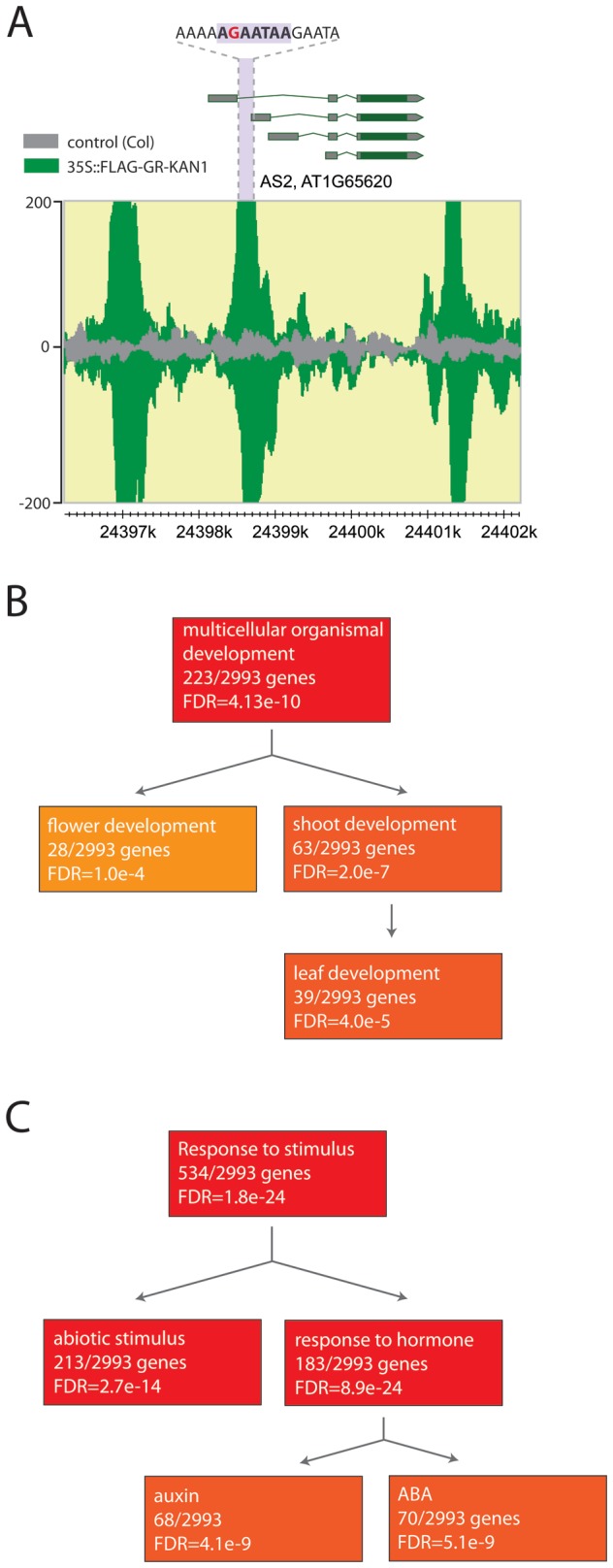
Gene-ontology analysis of KAN1 targets. **A**) KAN1 binds to the *ASYMMETRIC* *LEAVES2* (AS2) promoter. Three distinct binding regions were identified but only the second peak contains the VGAATAW motif. The guanine depicted in red is mutated to adenine in the *as2-5d* mutant. **B**) and **C**) Enrichment of GO terms identified in the set of genes located downstream of the KAN1-binding site. Over-representation of genes involved in multicellular organismal development and in the response to stimuli targeted by KAN1.

Next we examined whether our identified binding site is consistent with previous findings. The recently identified *as2-5d* mutation carries a G to A change in the promoter of *AS2*, causing ectopic *AS2* expression due to uncoupling from KAN1 regulation [31]. Our analysis revealed enrichment at three positions in the *AS2* promoter region previously identified to be recognized by KAN1. The sequence underlying the peak in the 5’ UTR of *AS2* contains the VGAATAW motif, with the G being exchanged for A in *as2-5d* (Figure 2A). This finding supports the idea that the 1802 binding regions containing the VGAATAW motif are recognized by KAN1 and represent genuine binding regions. Regions for which we can detect enrichment in our ChIP-Seq dataset which do not contain the VGAATAW motif might represent regions where KAN1 is associated to, maybe in complex with other DNA-binding proteins.

Taken together, we have developed an inducible system for KAN1 expression and used it to identify KAN1 binding sites across the *Arabidopsis* genome. Furthermore, we identified a *cis*-regulatory motif common to many of these targets that may represent a sequence directly recognized by KAN1.

### Promoters bound by KAN1

Having identified 1802 binding regions, we were interested in investigating whether genes encoding proteins with specific functions are enriched in this dataset. We therefore performed gene ontology studies using the Agrigo tool (http:// bioinfo.cau.edu.cn/agriGO/‎). This analysis revealed that genes with a function in multicellular organismal development are strongly over-represented in our dataset with further enrichment in the sub-categories flower development and shoot/leaf patterning (Figure 2B). Since KAN1 is a major patterning factor and our target gene analysis revealed an enrichment of other genes involved in patterning, this dataset contains genes with a high probability to be regulated by KAN1 (Dataset S2). In addition to genes with a role in development, we also identified genes whose products have known roles in responding to stimuli (Figure 2C). Of the hormonal signaling pathways, enrichment is found for genes involved in auxin and abscisic acid signaling supporting previous findings [30].

### Identification of genes transcriptionally regulated by KAN1

Having identified putative promoter regions bound by KAN1 using ChIP-seq, we next attempted to identify genes that respond transcriptionally to KAN1 activity. To this end, we utilized a line harboring a transgene resulting in widespread expression of a hormone inducible KAN1 protein, *35S:KAN1-GR* [6]. When seeds homozygous for the *35S:KAN1-GR* transgene were germinated in the presence of dexamethasone both shoot and root meristems were arrested, no leaf primordia were produced, and seedlings die a few weeks post germination, mimicking the phenotype of *35S:KAN1* plants [3]. As positive and negative controls we followed the expression of *AS2* and cyclophilin, respectively. When assayed 80 minutes after dexamethasone treatment, expression of *AS2* was reduced in hormone treated plants relative to controls, whereas cyclophilin expression was unchanged [10]. We next assayed genome-wide gene expression levels at two time points (80 minutes and 160 minutes) post-induction and identified 500 genes and 9 unannotated genomic regions in which gene expression was down-regulated at least at one of the time points (Dataset S3). In most instances down-regulation was observed at both time points, with 43 genes down-regulated only at 160 minutes and 4 genes down-regulated only at 80 minutes. Of the down-regulated genes, 42 are known to have a role in auxin biology (Dataset S4), including auxin transport or its regulation (*PIN1*, *PIN3*, *PIN4*, *PIN7*, *AUX1*, *PGP4*, *PGP19*, *PID*, *BIG*), auxin response (*IAA2*, *IAA3*, *IAA13*, *IAA14*, *IAA16*, *ARF4*, *ARF19*, *HAT2*), and auxin regulated genes (11 SAUR and 3 GH3 genes). Also down-regulated were 102 genes implicated in transcriptional regulation (Dataset S5), including some previously implicated in regulation of leaf polarity (e.g. *PHB*, *YABBY5*, *ARF4*). Some examples of each of these classes are shown in Figure S1. In contrast, up-regulation was detected at only 30 genes and 1 un-annotated region (Dataset S6). Since most potential target genes exhibited down-regulation, and KAN1 has been shown to interact with TOPLESS [38], a transcriptional co-repressor, we next identified genes that were both down-regulated and possessed local KAN1 binding sites.

### A set of putative KAN1 target genes identified through ChIP-seq are also transcriptionally regulated by KAN1

**Figure 3:**
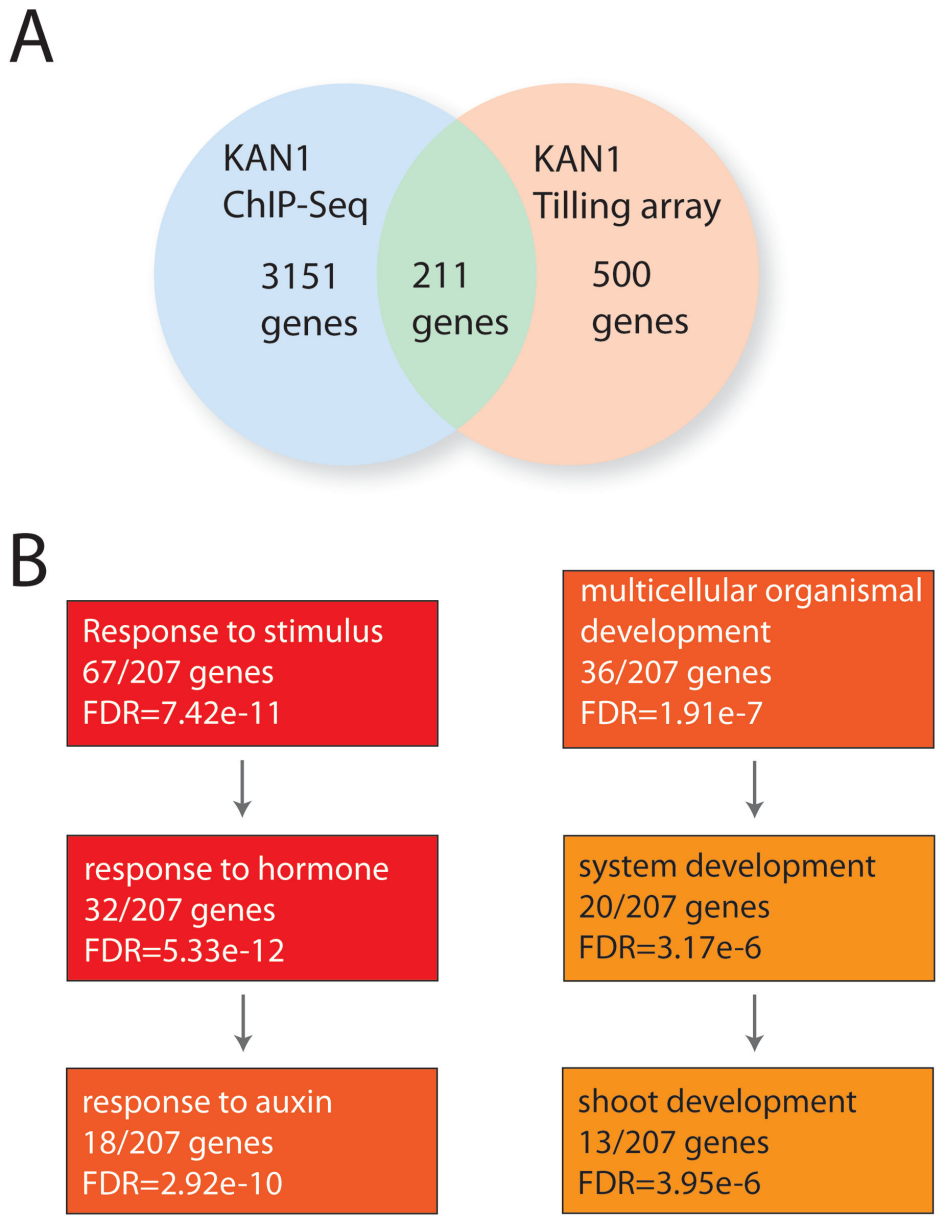
Genome-wide comparison of genes bound and regulated by KAN1. **A**) Venn-diagram showing numbers of genes bound by KAN1 and regulated by KAN1. The overlap contains 211 genes that are both bound and also regulated by KAN1. **B**) Gene ontology analysis of 211 potential direct KAN1 targets reveals a strong enrichment for genes involved in shoot patterning and the auxin response. Tables 1 and 2 contain these genes including the binding site information.

Among the 3151 putative KAN1 target genes selected from the ChIP-seq data analysis, a set of 211 genes was also regulated by KAN1 at 80 and/or 160 minutes post-induction (Figure 3A and Dataset S7) in the tiling array experiments. In addition, gene ontology classification of these ChIP-seq/tiling array overlapping genes revealed again a strong over-representation of genes involved in multicellular organismal development and response to stimulus, with a significant enrichment of genes involved in shoot development and auxin response, respectively (Figure 3B). Interestingly, among 19 genes related to organ development and shoot patterning (Table 1), four genes were previously shown to be important factors in the genetic network controlling organ patterning: *PHABULOSA* (*PHB*) and *ATHB8* (see also Figure S2), two class III HD-ZIP genes involved in the control of adaxial cell identity [1] and provascular patterning [1,39], respectively, *MIR166F*, which targets several *HD-ZIPIII* family members including *PHV*, *PHB*, *REV*, *ATHB-8* and *ATHB-15* [1,24,40], and *PIN-FORMED 1* (*PIN1*), an auxin efflux carrier required for organ formation and positioning [41-43]. Moreover, several genes such as *LONGIFOLIA 1* (*LNG1*) and *LNG2* [44], the BEL1-like homeodomain protein SAW2 [45], associated with leaf shape establishment, the receptor-like kinase PXY/TDR (PHLOEM INTERCALATED WITH XYLEM/TDIF RECEPTOR), involved in the proliferation of procambial cells as well as in the maintenance of polarity during vascular tissue development [46,47] , *NPY3*, *NPY5* (*naked pins in yuc mutants*) and the *PINOID* homolog *WAG2*, related to auxin-mediated organogenesis [48], were identified in both studies. Additional genes with a role in general aspects of shoot growth and development are listed in the Table 1.

**Table 1:** Potential KAN1 target genes with a role in organ or shoot development.

		**ChIP-seq data**							**Tiling array data**	
**AGI**	**Gene Symbol**	**ORP-rank**	**Distance**	**Location**	**Enrichment replicate 1**	**Enrichment replicate 2**	**FDR replicate 1**	**FDR replicate 2**	**80 min**	**160 min**	
AT1G13245	RTFL17	4140	2414	DOWN	4,3	2,7	8,20E-04	3,93E-04	yes	yes	
AT1G13260	RAV1	1215	519	DOWN	5,5	2,7	2,66E-39	2,20E-23	yes	yes	
AT1G13260	RAV1	1003	9835	UP	7,8	3,4	6,93E-49	1,03E-23			
AT1G13260	RAV1	882	6034	UP	5,1	2,8	1,54E-43	1,79E-33			
AT1G27320	AHK3	5004	630	DOWN	4,3	2,1	8,20E-04	1,29E+02	yes	yes	
AT1G56010	NAC1	7940	1500	UP	4,1	2,0	2,30E+04	1,80E+07	yes	yes	
AT1G73590	PIN1	1344	1049	DOWN	6,9	3,6	3,37E-34	8,69E-23	-	yes	
AT1G78240	TSD2	3471	2653	UP	5,0	2,5	2,67E-11	4,39E-04	yes	yes	
AT1G78240	TSD2	6421	3459	UP	6,0	2,0	4,37E-03	1,80E+07			
AT1G78240	TSD2	866	5964	UP	12,8	6,1	3,27E-49	1,04E-29			
AT1G78240	TSD2	3094	7048	UP	6,7	2,6	1,98E-19	1,28E-02			
AT2G23760	SAW2	1267	436	UP	7,2	3,8	9,78E-36	5,35E-25	-	yes	
AT2G23760	SAW2	1145	2496	DOWN	6,0	3,2	7,38E-38	5,57E-27			
AT2G31070	TCP10	344	76	UP	8,0	4,3	6,40E-73	1,79E-54	yes	yes	
AT2G34710	PHB	231	937	UP	10,2	4,2	1,91E-106	2,01E-52	yes	yes	
AT3G14370	WAG2	3554	1208	UP	7,9	3,5	1,08E-12	1,58E-02	yes	yes	
AT5G60970	TCP5	1164	2282	DOWN	7,3	4,4	1,15E-32	4,35E-30	yes	yes	
AT5G60970	TCP5	1019	3044	DOWN	7,8	4,9	2,90E-34	6,91E-34			
AT5G43603	MIR166F	2366	658	UP	3,8	2,7	2,25E-12	6,47E-18	yes	yes	
AT5G61480	PXY	2404		in CDS	5,7	2,6	5,67E-22	7,40E-09	-	yes	
AT5G67440	NPY3	434	413	UP	6,4	3,5	2,32E-65	1,24E-49	yes	yes	
AT5G67440	NPY3	1623	184	DOWN	5,5	2,5	1,19E-32	2,38E-16			
AT4G37590	NPY5	979	1452	UP	9,9	5,0	2,37E-43	3,80E-29	yes	yes	
AT3G02170	LNG2	3204	3955	UP	5,3	2,9	3,08E-11	6,67E-07	yes	yes	
AT5G15580	LNG1	4944		in CDS	10,7	2,6	1,57E-12	3,27E+06	yes	yes	
AT5G61960	AML1	2123	2369	UP	5,6	1,4	3,33E-34	4,59E-06	yes	yes	
AT5G61960	AML1	2265		in CDS	6,8	3,1	5,15E-23	3,05E-10			
AT4G32880	ATHB8	1402	561	UP	1,8	6,5	1,69E-30	3,33E-23	yes	yes	
AT2G46685	MIR166A	4624	3218	UP	2,3	4,8	4,90E-07	1,49E+02	-	-	
AT1G65620	AS2	1750		in CDS	2,4	5,1	1,50E-34	6,53E-12	-	-	
AT1G65620	AS2	2506	424	DOWN	2,4	5,9	1,72E-23	2,74E-06	-	-	
AT5G16560	KAN1	1996	173	UP	1,8	4,5	2,88E-21	5,12E-16	-	-	
AT5G16560	KAN1	2689	707	UP	2,6	9,3	8,92E-23	1,02E-04	-	-	
AT5G16560	KAN1	2930	5046	UP	2,9	7,8	8,53E-23	4,06E-03	-	-	
AT1G32240	KAN2	468	4442	DOWN	8,8	5	4,95E-61	4,17E-50	-	-	

Notes: By analyzing the ChIP-seq and the tiling array datasets and based on gene ontology (GO) analysis and literature contrast, we identified 23 genes involved in multicellular organismal development and shoot development. These genes are listed with the AGI (Arabidopsis Genome Initiative) gene code, the Gene Symbol, the ORP-rank, the distance from the binding site to the CDS, the location (UP=upstream of a gene, DOWN=downstream of a gene, in CDS), the enrichment of ChIP-seq replicates 1 and 2 (ratio of number of reads for a binding site in KAN1+DEX versus Col0+DEX), the false discovery rate (FDR) of ChIP-seq replicates 1 and 2, and the down-regulation at 80 and/or 160 min after KANADI1 activity induction (yes=the entire length of the predicted transcript was down-regulated; - no significant down-regulation).

Out of 211 genes identified as putative KAN1 targets by both the ChIP-seq and the tiling array approaches, 21 are involved in auxin response. Table 2 shows a set of KAN1 target genes encoding proteins involved in auxin signaling as well as in auxin transport. This set of genes includes several early auxin-regulated genes with a role in auxin signaling pathways such as two *Aux/IAA* genes (*IAA2* and *IAA13*), which encode short-lived transcription factors that function as repressors of auxin response genes [49], three *GH3* genes (*DFL1*, *DFL2* and *WES1*), encoding acyl adenylate–forming isozymes that covalently modify indole-3-acetic acid (IAA) [50], and three *SAUR-like* genes (*AT1G19840*, *AT1G75590* and *AT2G21210*), which encode short-lived nuclear proteins involved in auxin signaling by interacting with calmodulin [51,52]. Furthermore, an *AUXIN RESPONSE FACTOR* gene, *ARF4*, was identified in both experiments while *ARF3/ETT* was identified only in our ChIP-seq data. *ARF4*, together with the redundant gene *ARF3/ETT* (*ETTIN*), act to promote abaxial identity in association with KAN or its downstream targets [20]. In addition, it has been shown that the negative transcriptional, post-transcriptional and epigenetic regulation of these *ARFs* by AS1 and AS2 is important for the establishment of early leaf adaxial/abaxial polarity [53]. Among this set of genes, we also found the class II HD-ZIP gene *HAT2*, which is an early auxin-inducible gene with opposite functions in regulating auxin-mediated morphogenesis in the shoot and root tissues [54]. In a previous study, we also showed that *HAT2* acts downstream of REV in the shade avoidance response [29]. Regarding those genes involved in auxin transport, two *PIN* genes, *PIN3* and *PIN4*, which are important for tropic growth of the root [55] and root patterning [56], respectively, as well as for creating local auxin gradients required for the establishment of primordia and organ development [41], were found in both studies. Furthermore, a phospholipase required for PIN protein trafficking to the plasma membrane in the root (phospholipase A2; PLA2A) [57], and the PINOID protein kinase (PID), which controls PIN polarity and mediates changes in auxin flow to create local gradients for patterning processes [58], were identified. Additionally, the auxin influx transporter AUX1 and the ATP-binding cassette transporter AtMDR1 found in both studies regulate root gravitropism, and photomorphogenesis and root development, respectively, by mediating auxin polar transport [59,60].

**Table 2:** Potential KAN1 target genes involved in auxin response.

		**ChIP-seq data**							**Tiling array data**	
**AGI**	**Gene Symbol**	**ORP-rank**	**Distance**	**Location**	**Enrichment replicate 1**	**Enrichment replicate 2**	**FDR replicate 1**	**FDR replicate 2**	**80 min**	**160 min**
AT1G19840	SAUR-like	2341	6906	DOWN	5,1	2,8	6,76E-18	1,05E-12	yes	yes
AT1G75590	SAUR-like	3037	1915	UP	5,8	2,5	2,21E-17	1,86E-04	yes	yes
AT2G21210	SAUR-like	157	149	UP	8,3	3,4	4,47E-119	9,21E-58	yes	yes
AT2G33310	IAA13	1242		in CDS	7,4	4,1	1,00E-33	1,71E-27	yes	yes
AT2G34650	PID	2130	3923	UP	5,7	2,8	6,51E-23	1,53E-12	yes	yes
AT2G38120	AUX1	158	10052	UP	7,3	4,2	3,04E-94	1,24E-79	yes	yes
AT3G23030	IAA2	165	171	UP	9,0	4,4	4,11E-103	3,88E-67	yes	yes
AT3G28860	ATMDR1	3763	3456	UP	5,0	2,2	1,49E-11	1,45E-01	yes	yes
AT3G28860	ATMDR1	3075	8677	DOWN	4,4	2,0	3,47E-16	8,40E-05		
AT4G03400	DFL2	2385	2241	DOWN	7,4	3,1	9,71E-24	9,10E-08	yes	yes
AT4G03400	DFL2	1583	2654	DOWN	5,6	2,6	2,76E-33	1,08E-16		
AT4G27260	GH3.5, WES1	2996	1074	UP	4,0	2,2	6,37E-13	1,14E-07	yes	yes
AT4G27260	GH3.5, WES1	5906	971	DOWN	5,0	1,3	4,55E-09	1,63E+07		
AT5G47370	HAT2	3245	86	UP	4,6	2,3	1,64E-12	1,76E-05	yes	yes
AT5G54510	GH3.6, DFL1	2785	2222	DOWN	4,8	1,9	1,29E-22	1,16E-04	yes	yes
AT5G59780	MYB59	1240	5918	UP	5,6	3,3	2,16E-32	7,10E-28	yes	yes
AT5G61420	MYB28	1759	769	UP	6,2	2,7	5,59E-32	2,11E-13	yes	yes
AT5G61420	MYB28	1753	2117	UP	4,4	2,7	4,69E-22	6,78E-21		
AT5G63160	BT1	16	1273	UP	9,1	4,7	2,98E-185	8,71E-136	-	yes
AT5G67300	ATMYB44	2070	381	UP	4,5	2,4	1,11E-21	1,87E-14	yes	yes
AT5G67300	ATMYB44	53	2438	UP	11,3	5,4	9,41E-140	1,06E-92		
AT1G70940	PIN3	5237	20	UP	6,4	3,2	2,54E-03	2,51E+02	yes	yes
AT2G01420	PIN4	1622	367	UP	6,3	2,8	3,70E-35	3,35E-15	yes	yes
AT2G01420	PIN4	1630	973	DOWN	5,5	3,0	1,65E-27	7,06E-20		
AT2G26560	PLA2A	2249	1401	UP	6,1	3,1	1,59E-20	5,88E-12	yes	yes
AT5G60450	ARF4	294	2112	UP	6,7	4,0	6,92E-70	1,80E-64	yes	yes
AT2G33860	ARF3/ETT	4041	1151	UP	3,9	3,1	6,30E-01	2,50E-07	-	-
AT1G15690	AVP1	3612	2238	UP	8,1	3,4	4,21E-13	8,87E-02	yes	yes

Notes: By analyzing the ChIP-seq and the tiling array datasets and based on gene ontology (GO) analysis and literature contrast, we identified 22 genes involved in response to auxin. These genes are listed with the AGI (Arabidopsis Genome Initiative) gene code, the Gene Symbol, the ORP-rank, the distance from the binding site to the CDS, the location (UP=upstream of a gene, DOWN=downstream of a gene, in CDS), the enrichment of ChIP-seq replicates 1 and 2 (ratio of number of reads for a binding site in KAN1+DEX versus Col0+DEX), the false discovery rate (FDR) of ChIP-seq replicates 1 and 2, and the down-regulation at 80 and/or 160 min after KANADI1 activity induction (yes=the entire length of the predicted transcript was down-regulated; - no significant down-regulation).

Finally, several genes previously described as being involved in adaxial/abaxial patterning of the leaf and the vascular tissues such as *MIR166A* and *AS2* [1,17,18,24,40] were identified as KAN1 targets exclusively through the ChIP-seq approach. Moreover, *KAN1* itself and *KAN2* were isolated as putative targets suggesting that KAN1 may control its own expression as well as the expression of other KAN gene family members.

### Genes oppositely regulated by the REV/KAN1 module

REV and KAN1 have opposite functions in early leaf patterning. In order to determine whether the antagonistic roles can be attributed to an opposite regulation of common downstream target genes, we compared potential downstream REV target genes identified by ChIP-Seq [29] with the list of genes bound and regulated by KAN1 (Dataset S7). This analysis resulted in the identification of 26 genes, which are candidates for dual regulation (Table 3). Interestingly, five genes are bound by REV and KAN1 in a region less than 100bp apart, suggesting that, besides dual regulation, REV and KAN1 might also compete for chromatin accessibility. All five genes (TEM, ZFP4, SUC1, a receptor protein kinase and a NPH3-like protein) seem to be involved in the control of development corroborating the idea that they act downstream of developmental regulators. 

**Table 3:** Genes potentially cross-regulated by the REV/KAN1 module.

		**REV ChIP-seq**		**KAN1 ChIP-seq**						
**AGI**	**Gene symbol**	**Distance**	**Location**	**FDR replicate 1**	**FDR replicate 2 **	**Enrichment replicate 1**	**Enrichment replicate 2**	**Distance**	**Location**	**Distance REV/KAN1 binding**
AT1G22570	Major facilitator protein	1129	DOWN	1,19E-112	3,31E-68	13,5	6,2	920	UP	4085
AT1G25560	TEM1	2926	UP	2,15E-64	2,70E-31	11,7	4,9	3024	UP	61
AT1G51940	LysM-domain protein	1525	UP	1,22E-10	3,24E+02	4,5	1,8	5999	UP	4504
AT1G61660	bHLH transcription factor		in CDS	2,26E-37	1,47E-22	6,8	3,4	1365	UP	3405
AT1G66140	ZFP4		in CDS	3,75E-31	4,10E-17	6,1	2,9		in CDS	26
AT1G66140	ZFP4			1,49E-01	1,35E+02	4,0	2,2		in CDS	434
AT1G67710	ARR11	1466	UP	2,20E-54	6,92E-40	6,3	3,4	1375	UP	113
AT1G68130	IDD14	3051	UP	1,08E-12	9,72E-11	3,8	2,2	3357	DOWN	8739
AT1G68520	B-BOX zinc finger protein	741	DOWN	4,34E-26	3,09E-09	3,8	2,3	248	UP	2757
AT1G68520	B-BOX zinc finger protein			5,97E-14	2,76E-06	5,9	2,8	829	UP	3408
AT1G71880	SUC1	5858	UP	2,80E-74	1,99E-51	8,1	4,1	5878	UP	41
AT1G72300	Leucine-rich receptor protein	3145	UP	4,42E-22	4,03E-12	8,3	4,1	691	UP	2424
AT1G72300	Leucine-rich receptor protein			6,72E-09	1,69E-09	4,0	2,4	1465	UP	1630
AT3G02140	TMAC2	893	UP	2,04E-69	5,94E-57	10,5	5,9	201	UP	653
AT3G02140	TMAC2			1,01E-48	5,34E-34	7,4	3,7	2737	UP	1863
AT3G02140	TMAC2			4,50E-08	5,09E-03	4,8	2,5	3049	UP	2231
AT3G12920	BRG3	1579	DOWN	1,73E-04	8,92E-01	4,3	2,3	1782	UP	5019
AT3G12920	BRG3			1,22E-181	7,71E-141	13,2	7,0	4565	UP	7652
AT3G12920	BRG3			6,75E-90	8,25E-62	11,6	5,8	983	DOWN	568
AT3G12920	BRG3			1,13E-63	9,51E-49	9,9	5,4	2338	DOWN	797
AT3G15570	NPH3 family protein	1009	UP	6,12E-93	1,02E-63	8,9	4,4	1036	UP	14
AT3G54400	Aspartyl protease protein	602	UP	4,51E+02	8,26E+04	4,6	2,4	120	UP	402
AT3G56050	Protein kinase family protein	208	UP	4,29E-87	5,13E-48	8,8	3,9	274	UP	42
AT3G61460	BRH1	2196	UP	1,85E-17	4,61E-12	5,9	3,2	314	UP	1826
AT4G18700	CIPK12	282	DOWN	3,22E+00	3,57E+03	7,5	3,9	26	UP	2468
AT4G18700	CIPK12			4,36E-101	1,53E-92	8,4	5,0	133	DOWN	146
AT4G22190	unknown protein	2827	UP	1,45E-38	9,85E-30	8,1	4,5	1709	UP	1166
AT4G26540	Leucine rich repeat receptor	2234	UP	3,06E-86	1,40E-45	8,5	3,7	2160	UP	129
AT4G26540	Leucine rich repeat receptor			8,31E-78	1,31E-53	7,1	3,6	896	DOWN	6893
AT4G27260	GH3.5, WES1	2494	DOWN	6,37E-13	1,14E-07	4,0	2,2	1074	UP	6295
AT4G27260	GH3.5, WES1			4,55E-09	1,63E+07	5,0	1,3	971	DOWN	1591
AT5G05690	CPD	4847	UP	1,81E-06	1,67E-02	5,1	2,7	5642	UP	894
AT5G47370	HAT2	1548	UP	1,64E-12	1,76E-05	4,6	2,3	86	UP	1403
AT5G51550	EXL3	2573	UP	2,22E-174	1,08E-139	14,1	7,7	687	UP	1892
AT5G51550	EXL3			9,95E-19	1,35E-11	4,0	2,1	2133	UP	480
AT5G52060	ATBAG1, BAG1	739	UP	1,28E-20	2,48E-03	5,4	2,1	8	UP	745
AT5G64570	XYL4	2389	UP	1,84E-39	5,08E-19	4,9	2,2		in CDS	5597
AT5G64570	XYL4			5,70E-50	5,28E-46	7,7	4,6	331	UP	2090
AT5G67190	DEAR2	2710	UP	1,46E-33	1,01E-22	4,9	2,6	276	UP	2557
AT5G67190	DEAR2			5,02E-178	3,45E-151	14,2	8,1	1541	UP	1248
AT5G67190	DEAR2			1,28E-61	2,35E-45	6,9	3,6	1319	UP	6884

Notes: By comparing the REV target genes identified by ChIP-Seq [29] with the list of genes bound and regulated by KAN1 (Dataset S7), we identified 26 genes which are candidates for dual regulation. These genes are listed with the AGI (Arabidopsis Genome Initiative) gene code, the Gene Symbol, the false discovery rate (FDR) of ChIP-seq replicates 1 and 2, the enrichment of ChIP-seq replicates 1 and 2 (ratio of number of reads for a binding site in KAN1+DEX versus Col0+DEX), the distance from the binding site to the CDS and the location (UP=upstream of a gene, DOWN=downstream of a gene, in CDS), and the distance between REV and KAN1 binding sites.

## Discussion

In this study, we utilize inducible overexpression of *KAN1* to identify KAN1 responsive genes and direct targets. Although such an approach may lead to artifacts because of the ectopic and artificially high expression levels used, the set of genes we have identified shows enrichment for genes involved in development and auxin biology, suggesting our experiments have identified genes that are biologically relevant.

Our results show that the VGAATAW motif may be a common *cis*-regulatory element recognized by KAN1, which includes the motif affected by the *as2-5d* point mutation that causes ectopic *AS2* expression due to its regulation being uncoupled from KAN1 [31]. We have focused our attention on the 1802 binding regions containing this motif (corresponding to 3151 genes potentially regulated by KAN1) and, especially, on those genes that exhibit gene expression changes in response to induction of KAN1 activity. Several of the identified downstream targets have a role in organ development, shoot patterning or auxin response and transport. In addition, we present a set of genes that are potentially controlled by both KAN1 and REV. The potential regulation of the selected genes by KAN1 and its link with patterning processes and auxin-related events as well as the gene regulation by the module KAN1/REV are discussed below.

### KAN1 regulates many genes related to organ patterning

In our study, we find that KAN1 binds to the promoter of two *HD-ZIPIII* genes, *PHABULOSA* and *ATHB8* (Table 1) and represses their expression, suggesting that both *HD-ZIPIII* genes are direct targets of KAN1 during organ polarity establishment. In previous studies, it was proposed that the antagonistic role between KAN and HD-ZIPIII activities in vascular tissue formation is mediated by affecting the canalization of auxin flow rather than through a direct interaction between both families of transcription factors [10]. However, our results suggest that there may be contexts in which KAN1 acts directly on *PHB* and *ATHB8*. 

We also find that KAN1 binds directly to the promoters of *MIR166A* and *MIR166F* and down-regulates the expression of *MIR166F* (Table 1), suggesting that at least KAN1 may directly regulate *MIR166F*. In addition, our results indicate that KAN1 binds to its own promoter and *KAN2* via the VGAATAW motif (Table 1) but also potentially KAN3 (Dataset S1), although no VGAATAW motif was found for this binding event. Taken together, these results suggest that in some contexts KAN1 may direct a negative feedback loop that limits the levels of several abaxial factors including KAN1 itself. 

KAN1 binds to the proximal promoters and represses the expression of genes involved in different aspects of organ development such as PXY/TDR, *LNG1*/*2* and *SAW2* (Table 1). Like *KAN1*, *PXY* is a key gene in vasculature polarity establishment. In particular, *PXY* is required for the proper orientation of cell divisions in the vascular meristem, which gives rise to specialized and spatially separated xylem and phloem cells [46,47]. The homologous genes *LNG1* and *LNG2* regulate leaf morphology by positively promoting longitudinal polar cell elongation [44]. The adaxial epidermal cells of the midveins and the leaf blade are longitudinally elongated in the *lng1-1D* mutant plants compared with wild type. *SAW2* controls leaf shape and exhibits adaxial expression in developing lateral organs [45]. Therefore, our results suggest that KAN1 may directly regulate genes involved in the development of lateral organs and vascular tissue, known sites of KAN1 activity. 

### KAN1 regulates auxin-related genes

Organ patterning is in part modulated by the polar transport of auxin to specific locations, generating auxin maxima that promote organ initiation and growth. PIN proteins play an important role in the regulation of auxin distribution. Loss of proper PIN polarity establishment, as in PIN multiple mutants and *gn* mutants, leads to embryo patterning defects [61-65]. Previous studies have shown a negative effect of KAN1 on PIN1 activity. Thus, ectopic expression of *PIN1* is observed in *kan1 kan2 kan4* embryos, suggesting that KAN genes may act to restrict auxin flow during embryogenesis by regulating *PIN1* gene expression [7]. *PIN1* gene expression alterations have also been observed at the ectopic abaxial leaf outgrowths of *kan1 kan2* plants. In particular, *PIN1* expression was higher in the outgrowths than in the surrounding leaf tissue, suggesting that the outgrowths may be due to ectopic auxin maxima forming in the lamina [3,4]. In addition, it has been shown that polar auxin flow is essential to form procambium cells in vascular tissues, and KAN genes play a role in the distribution of this auxin flow by restricting PIN1 activity [10]. In agreement with these findings, we have identified a binding site for KAN1 downstream from *PIN1* that likely mediates direct repression of *PIN1* by KAN1 (Table 1). Motifs adjacent to other *PIN* genes such as *PIN3* and *PIN4* were also bound by KAN1, and their expression was repressed by KAN1 as well (Table 2). Therefore, KAN1 may directly regulate several PIN family members supporting previous findings that showed that, at least in some contexts, KAN proteins may act in patterning processes through auxin transport modulation. Additionally, and reinforcing this hypothesis, KAN1 bound and repressed several genes involved in the regulation of PIN activity and trafficking such as *PINOID* and *PLA2A* [57,58,66], respectively. *NPY3*, *NPY5* and *WAG2*, which are thought to act together to determine what side of the cell PIN accumulates at [48,67-69], and genes involved in auxin polar transport such as the auxin influx transporter *AUX1* and the ATP-binding cassette transporter *AtMDR1* [59,60] were also bound and repressed by KAN1 (Table 2). 

Our data also point to a direct effect of KAN1 on auxin signaling pathways. For instance KAN1 bound near and repressed early auxin response genes including three *GH3* genes (*DFL1*, *DFL2* and *WES1*), three *SAUR-like* genes (*AT1G19840*, *AT1G75590* and *AT2G21210*) as well as two *Aux/IAA* genes (*IAA2* and *IAA13*) [49,70] (Table 2).

A connection of auxin signaling comes in addition from direct repression of *ARF4* and binding of *ARF3* by KAN1 (Table 2). The phenotype of *ett arf4* leaves resembles the phenotype of *kan1 kan2* leaves, leading to the proposal that ARF proteins act together with KAN proteins or its downstream targets to regulate transcription [20]. While this previous study suggested a positive interaction between these transcription factors, our findings suggest that there may also be negative feedback between *KAN1* and *ARF4* and *ARF3/ETT*, potentially again (see above), as a mechanism to maintain homeostasis among factors controlling abaxial identity. 

We have identified several additional genes involved in auxin transport and its regulation or auxin signaling as being repressed after *KAN1* induction and, in some cases, also bound by KAN1 (Dataset S1 and Figure S1). This set of genes includes *PIN7*, which is involved in apical–basal axis formation of the embryo [62], *YABBY5*, a transcription factor involved in abaxial cell fate specification and auxin distribution [21,23], different early auxin-responsive genes such as *GH3.3*, the SMALL AUXIN UP RNAs (SAUR) *SAUR19*, *SAUR20* and *SAUR63*, which regulate auxin polar transport and promote auxin-mediated organ elongation [71,72], three SAUR-like genes (*AT1G19840*, *AT4G38840* and *AT5G18030*), *ARF19*, *IAA3*, *IAA16*, *IAA14*, and an auxin receptor belonging to the TIR1 subfamily (*AFB1*) that interacts with Aux/IAA proteins [73,74]. In addition, in a previous study [29], we demonstrated that the expression of *HAT2*, which was also bound and repressed by KAN1 in the current study, and two genes that encode auxin biosynthetic enzymes, *TAA1* and *YUC5*, is reduced significantly after KAN1 induction. These results together with our findings reflect that, certainly, KAN1 may control the influence of auxin on organ development through complex interactions and at different levels: biosynthesis, transport and its regulation, and signaling.

Finally, our results suggest that KAN1 may act on other hormone pathways through the regulation of genes involved in the response to abscisic acid, jasmonic acid, brassinosteroids, ethylene, cytokinins and gibberellins (Dataset S2 and Figure S1).

### Regulation by KAN1 and REV of common downstream target genes

Genetic analysis has indicated that the HD-ZIPIII and KAN factors act oppositely in organ patterning [1,3,7]. However, it remains unclear whether this interaction occurs by direct mutually antagonistic regulation, through opposing regulation of a set of common direct targets or through opposing regulation of indirect targets. With respect to direct antagonistic regulation, in the current study, we did not find evidence of direct regulation of *REV* by KAN1, although KAN1 appears to bind other HD-ZIPIII genes such as *PHB* and *ATHB8* and to repress their expression. On the other hand, published work identifying *HAT2*, *TAA1* and *YUC5* as genes oppositely regulated by REV and KAN [29] supports the shared common targets hypothesis. To further investigate whether REV and KAN1 act on additional common target genes, we compared the ChIP-Seq data for KAN1 with those recently obtained for REV [29] and found an additional set of overlapping putative target genes that bring the total to 26 genes (Table 3). Among these, we found genes encoding transcription factors and proteins involved in hormone-associated processes. Finally, several genes involved in auxin transcriptional response and auxin transport are repressed by KAN1, whereas auxin biosynthesis and transport are positively regulated by HD-ZIPIII activity. Thus, another mechanism by which KAN1 and HD-ZIPIII activities have opposing effects is via antagonistic regulation of auxin biology, which does not necessarily occur at the level of the same transcriptional targets but will create steep auxin gradients that could function as positional signals.

The vast majority of KAN1 targets identified were down-regulated. Together with the observations that KAN1 directly represses the expression of the adaxial factor *AS2* [31] and that TOPLESS, a co-repressor protein, directly interacts with KAN1 [38], our data suggests that KAN1 primarily acts as a repressor. According to the opposite regulation of common targets hypothesis, if KAN1 acts as a repressor, the HD-ZIPIII proteins should act as activators of those genes that are common targets. Consistent with HD-ZIPIII proteins acting as activators, expression of REV translationally fused with a repressor domain (REV-SRDX) phenocopies *phb phv rev* plants (Dyani Lewis and J. L. Bowman, unpublished data). Thus, our findings together with published work [1,3,7,29,31,38] indicate that HD-ZIPIII and KAN genes function antagonistically both through mutual regulation as well as through the opposite regulation of common direct targets and indirect targets. Mutual regulation may ensure the proper partitioning of adaxial and abaxial tissues while the opposite regulation of common targets may help set up contrasting transcriptional activities that distinguish adaxial and abaxial cell types.

## Supporting Information

Dataset S1 **All ChIP-Seq identified regions containing the VGAATAW element.**
(XLS)

Dataset S2 **Putative KAN1 targets with roles in system and organ development and hormone signaling.**
(XLS)

Dataset S3 **All genes down-regulated by KAN1.**
(XLS)

Dataset S4 **Genes involved in auxin biology down-regulated by KAN1.**
(XLS)

Dataset S5 **Genes involved in transcriptional regulation down-regulated by KAN1.**
(XLS)

Dataset S6 **All genes up-regulated by KAN1.**
(XLS)

Dataset S7 **Overlap ChIP-Seq/tiling array.**
(XLS)

Figure S1 **Examples of raw tiling array data.** The lower two lines in each figure represent the 80 minute time point and the upper two lines represent the 160 minute time point. The upper of the lines in each time point are from a single biological experiment, whereas the lower are the average from two biological replicates. Genes are identified by their AtNg and common names, and those genes that were detected as also bound by KAN1 are denoted by an *.
(TIF)

Figure S2 **Genes bound by KAN1 are also regulated by KAN1 at the transcriptional level.** **A**) ChIP-Seq graphs show enrichment for KAN1 binding in the 3’ region of the ATHB8 gene. The enriched region contains the VGAATAW motif. **B**) *ATHB8* expression is strongly repressed in DEX-treated *35S::FLAG-GR-KAN1* transgenic plants. Plotted are relative qRT-PCR expression values of two independent biological replicates. Each biological experiment was carried out with four technical replicates and average values with standard deviation were calculated. *p≤0.01; **p≤1.0E-06.
(TIF)
