# Supplementary figures and images for: Genome-Wide Identification of KANADI1 Target Genes
Source: PLoS One. 2013 Oct 14;8(10):e77341. doi: 10.1371/journal.pone.0077341 (PMC3796457; doi:10.1371/journal.pone.0077341)

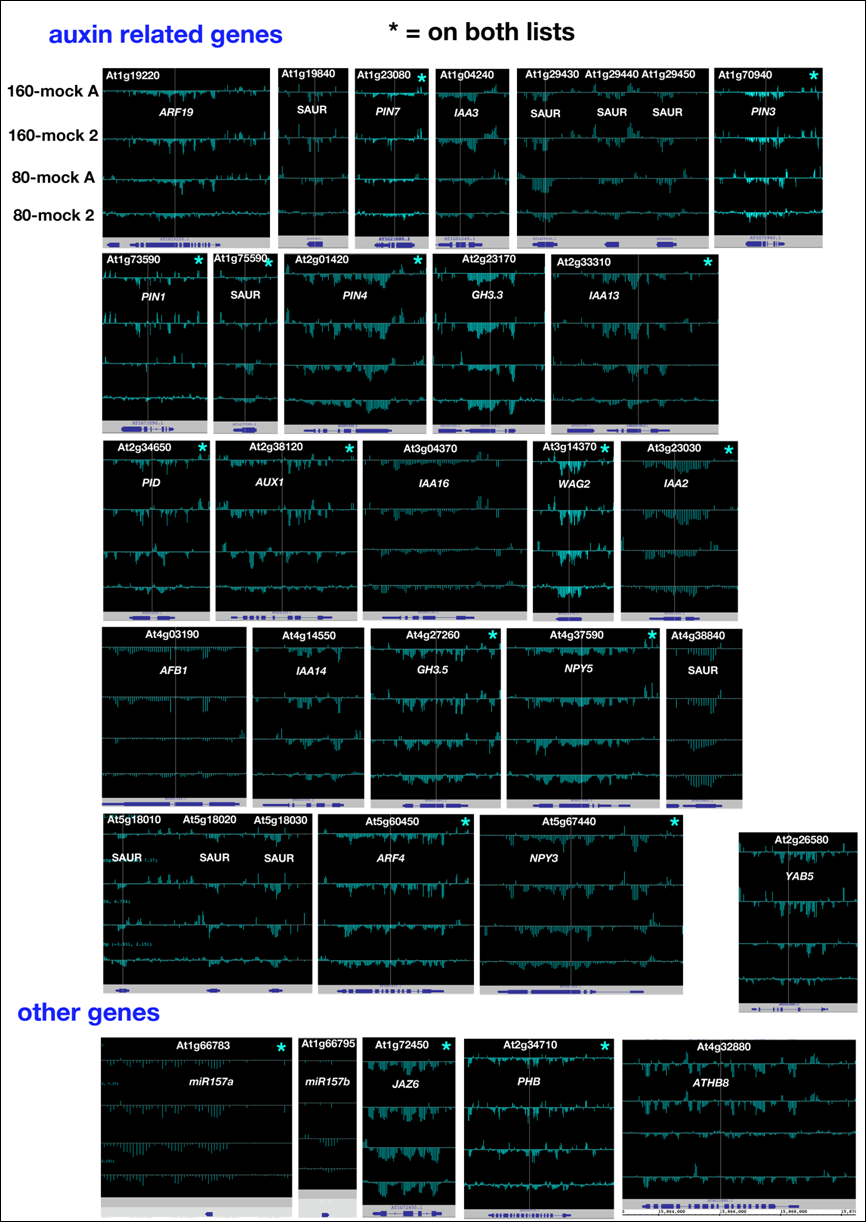

Supplement: Figure S1 — Examples of raw tiling array data. The lower two lines in each figure represent the 80 minute time point and the upper two lines represent the 160 minute time point. The upper of the lines in each time point are from a single biological experiment, whereas the lower are the average from two biological replicates. Genes are identified by their AtNg and common names, and those genes that were detected as also bound by KAN1 are denoted by an *. (TIF) [file pone.0077341.s008.tif]

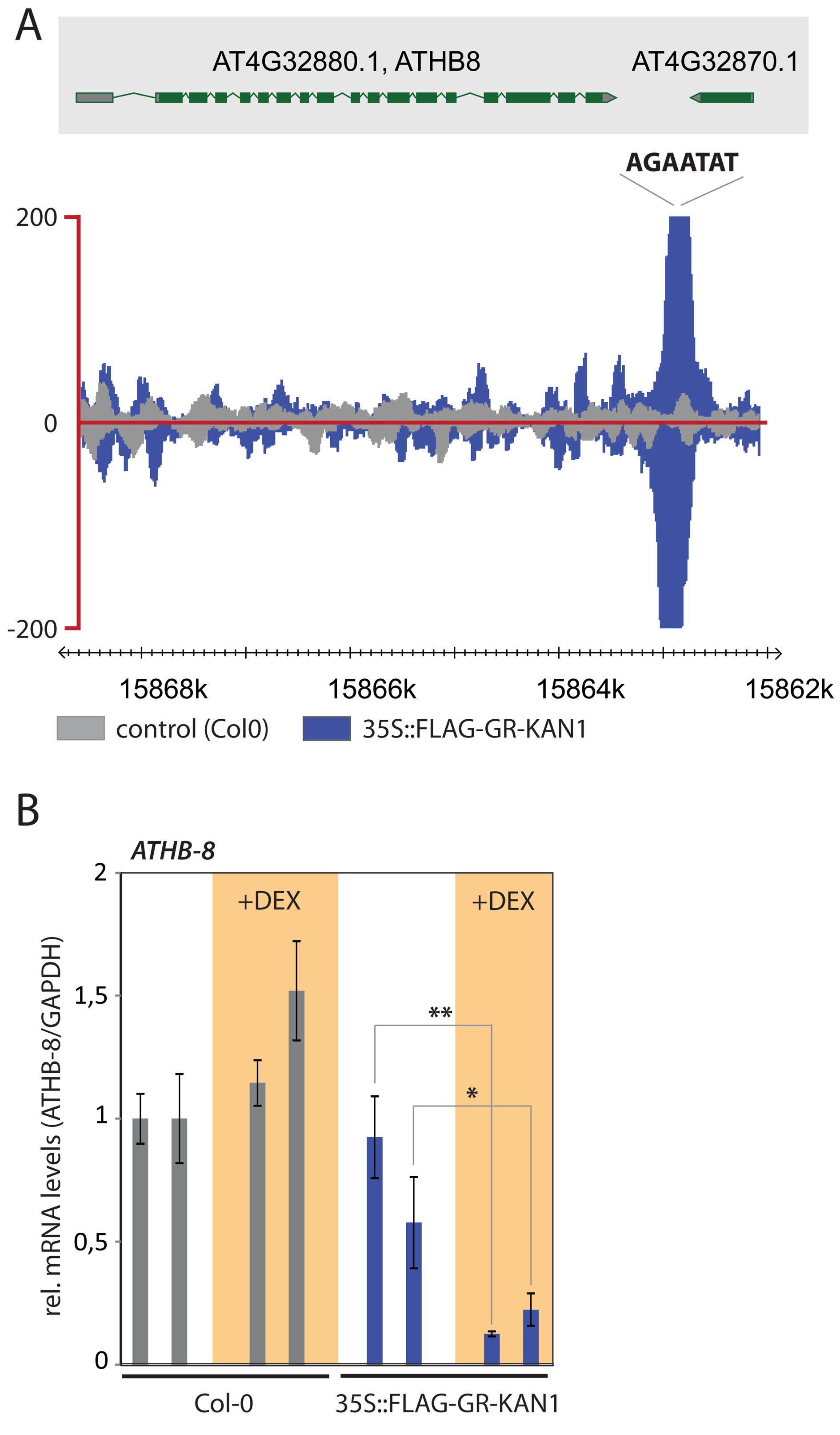

Supplement: Figure S2 — Genes bound by KAN1 are also regulated by KAN1 at the transcriptional level. A) ChIP-Seq graphs show enrichment for KAN1 binding in the 3’ region of the ATHB8 gene. The enriched region contains the VGAATAW motif. B) ATHB8 expression is strongly repressed in DEX-treated 35S::FLAG-GR-KAN1 transgenic plants. Plotted are relative qRT-PCR expression values of two independent biological replicates. Each biological experiment was carried out with four technical replicates and average values with standard deviation were calculated. *p≤0.01; **p≤1.0E-06. (TIF) [file pone.0077341.s009.tif]
